# Stages in the catalyst-free InP nanowire growth on silicon (100) by metal organic chemical vapor deposition

**DOI:** 10.1186/1556-276X-7-321

**Published:** 2012-06-20

**Authors:** Guoqing Miao, Dengwei Zhang

**Affiliations:** 1 State Key Laboratory of Luminescence and Applications, Changchun Institute of Optics, Fine Mechanics and Physics, Chinese Academy of Sciences, Changchun, 130033, People’s Republic of China

**Keywords:** InP, nanowire, catalyst-free growth, MOCVD, photoluminescence, Raman

## Abstract

Catalyst-free InP nanowires were grown on Si (100) substrates by low-pressure metal organic chemical vapor deposition. The different stages of nanowire growth were investigated. The scanning electron microscopy images showed that the density of the nanowires increased as the growth continued. Catalyzing indium droplets could still be fabricated in the nanowire growing process. X-ray diffraction showed that the nanowires grown at different stages were single crystalline with <111 > growth direction. The photoluminescence studies carried out at room temperature on InP nanowires reveal that the blueshift of photoluminescence decreased as the growing time accumulates, which is related to the increase in the diameter, rather than the length. Raman spectra for nanowires at different growing stages show that the quality of the nanowire changes. The growth of InP nanowires at different growing stages is demonstrated as a dynamic process.

## Background

Semiconductor nanowires of different materials have received increasing attention in recent years for their nanodevice application as one-dimensional structures and building blocks [1-6]. III-V nanowires grown on Si substrate have superior electronic properties of direct-bandgap III-V materials combined with the low cost and well-known properties of silicon. One way of fabricating III-V nanowires is by vapor–liquid–solid (VLS) method [7] using metal organic chemical vapor deposition (MOCVD) [8]. In the typical fabrication of III-V semiconductor nanowires by the VLS method, gold nanoparticle has been used as catalyst [9]. However, gold nanoparticle-mediated growth produces unwanted effects in nanowires, such as introduction of deep levels [10] and gold migration [11] on the semiconductor surface. In order to avoid these impacts, catalyst-free growth is demonstrated by using *in situ* deposited indium droplets as seeds for nanowire growth [12].

Catalyst-free InP nanowire growth has been realized by MOCVD, and the influence of different growing conditions has been discussed [13]. Vertical InP nanowires have been grown on Si (111) using indium droplets as the catalysts [14]. Individual bulk-like wires of wurtzite InP have been investigated by photoluminescence, photoluminescence excitation spectroscopy, and transmission electron microscopy [15]. Size-dependent photoluminescence from single indium phosphide nanowires has been discussed [16]. If production of nanowires is to reach a technologically relevant scale, exact control of the structure is necessary, and this entails a greater understanding and control of the growth process. In this paper, we study the entire forest of InP nanowires at different growing stages during the MOCVD growth process for a single set of growth conditions. In the following, we first present that new nanowires could grow in a catalyst-free InP nanowire growing process. We then proceed to describe the nanowires during different stages of the growth process by X-ray diffraction. Finally, we identify that the photoluminescence (PL) of the nanowires has direct relations with the diameter and the crystal quality of InP nanowires at different growing stage changes.

## Methods

InP nanowires were grown by low-pressure MOCVD at the pressure of 10 kPa, with trimethylindium (TMIn) and phosphine (PH_3_) as precursor materials, transported in H_2_ as carrier gas. For In droplet fabrication, TMIn was introduced to the reactor at a mole flux of 4.78 × 10^−6^ mol/min for 30 s at 330 °C. Then, PH_3_ was introduced into the reactor immediately to start the nanowire growth. The nanowire was grown at 330 °C. For the precursors, the mole flux of TMIn is 4.78 × 10^−6^ mol/min and that of the PH_3_ is 1.4 × 10^−4^ mol/min; the V/III ratio for nanowire growth is 30. After the growth was completed, TMIn was turned off and PH_3_ was continuously inputted to protect the substrate until the temperature drops to 250 °C. The time for nanowire growth changed from 30 s, 1 min, 2 min, 3 min, 5 min, 7 min, till 15 min. The InP nanowires were characterized by scanning electron microscopy (SEM), X-ray diffraction, PL, and Raman. For PL, the 532-nm wavelength of a laser and a liquid N_2_-cooled Ge detector were used. The 488-nm wavelength of a laser is used as excitation source for Raman diffraction.

## Results and discussion

**Figure 1:**
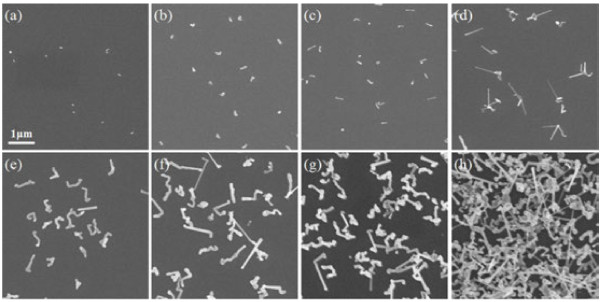
**SEM images of In droplets and grown nanowires.** (**a**) In droplets. Nanowires grown for (**b**) 30 s, (**c**) 1 min, (**d**) 2 min, (**e**) 3 min, (**f**) 5 min, (**g**) 7 min, and (**h**) 15 min. The scale bar is 1 μm and applies for all images.

Top-view images of indium catalyst droplets and InP nanowires at different growing stages are presented in Figure 1. It clearly shows that the density of the nanowires increases as the growing time gets longer. This phenomenon will not appear in the traditional Au-catalyzed nanowire MOCVD growth, for the density of the gold droplets is certain as soon as they were fabricated in the pretreatment. Thus, the density of the nanowires grown on Au droplets is certain. The catalyst-free InP nanowire growth is divided into two steps: catalyst fabrication and nanowire growth. Nanowires grow on the base of the indium droplets fabricated in the front step. The density of the nanowires increases as the time accumulates, which means that besides the nanowires grown on the catalyst, the new nanowires are fabricated in the nanowire growing process.

**Figure 2:**
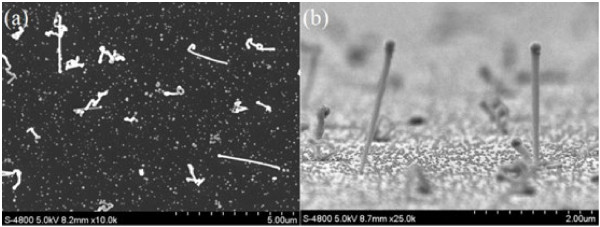
**SEM images of InP nanowires grown for 10 min without indium droplet fabrication before nanowire growth.** (**a**) Top-view image. (**b**) Lateral-view image.

In order to verify whether new nanowires could grow in the nanowire growing process, we cancel indium droplet fabrication and the Si substrate with no indium droplets is put in the circumstance of nanowire growth for 10 min. The corresponding SEM images of the sample are given in Figure 2. There are some nanowires and abundant pellets on the substrate. Figure 2b shows the nanowires on the substrate. There is a catalyst nanoparticle at the top, meaning that these nanowires are grown on the base of the catalyst. The two images indicate that, in the atmosphere of TMIn and PH3, indium droplets can still be fabricated and provide seeds for new nanowire growth.

There are two possible reasons for that. One is the physical condition especially the temperature. The catalyst indium droplets are fabricated by the thermal decomposition of TMIn. For catalyst-free InP nanowire growth, it has been tested in our experiments before that the temperature is in the range of 330 °C to 370 °C [17,18]. In this range, indium droplets can be fabricated [19]. This provides physical possibility for indium droplet fabrication during the nanowire growing step.

The other is the TMIn on the surface of the substrate which provides material for the fabrication of new indium droplets. In the model of the nanowire growing process on the sidewall, the precursor material on the surface of the substrate can be absorbed by the nanowires in the way of adatom diffusion and every single nanowire can only absorb a limited area around itself [20]. Figure 1a shows that the catalyst droplet, which provides the initial seed for nanowire growth, is scatter distributed on the substrate and there is large space between the droplets. In the nanowire growing process, the TMIn in these spaces is decomposed and accumulates to fabricate indium droplets. These droplets are the seeds for new nanowire growth.

By comparing the images of Figure 1, it is found that the density of nanowires had almost no change in the first 3 min. After 5 min, the density obviously increased. This means that the indium droplets take more than 3 min to be fabricated, as the space and the material TMIn for indium droplet fabrication are not as sufficient as the catalyst-fabricating step. Since the density of the nanowires in Figure 2a is quite low, the new nanowires fabricated in the growing process take a limited part in the catalyst-free nanowire growth.

**Figure 3:**
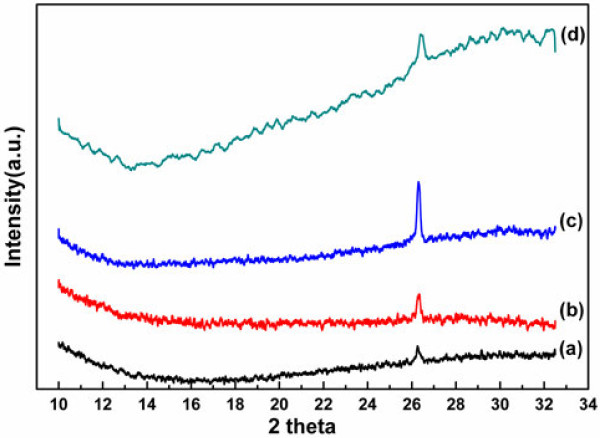
**X-ray powder diffraction pattern of the grown InP nanowires.** (**a**) 5, (**b**) 7, and (**c**) 15 min. (**d**) Nanowires grown for 10 min with no indium droplet fabrication.

The nanowires grown for less than 5 min can hardly be examined by X-ray diffraction as they are too short and their density is so low. As shown in Figure 3, the diffraction peaks of patterns (a) to (c) are all at 26.3°, proving that the nanowires grow along the <111 > direction. As the InP nanowires increase in unit area along with the growing time, the intensity of the diffraction peak rises from (a) to (c). The diffraction peak of the nanowires grown without a catalyst is at 26.3° too, indicating that the nanowires fabricated in the growing process are along the <111 > direction.

**Figure 4:**
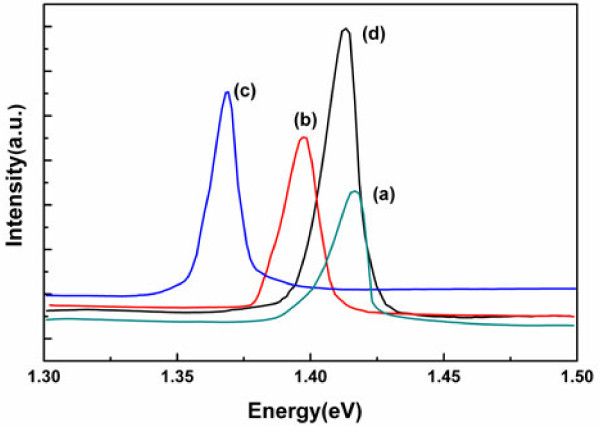
**Room-temperature PL spectra of InP nanowires grown for (a) 2, (b) 5, and (c) 7 min.** Trace (**d**) is for the nanowires grown for 10 min based on the smaller catalyst fabricated.

The optical properties of InP nanowires were evaluated by PL measurements at room temperature and shown in Figure 4. Nanowires grown for different times have different PL peaks. From traces (a) to (c), the PL intensity increases with the increasing time of the nanowire growth. This is due to the increase in dimension and density of the nanowires. The PL peaks are separately at 1.416, 1.397, and 1.368 eV, corresponding to the growing time of 2, 5, and 7 min, respectively. Compared to the bulk InP, there is blueshift which decreases as the growing time accumulates. It is found that the amount of the blueshift of PL is dependent on the time of the nanowire growth, which in turn is dependent on the dimensions of the nanowire.

The blueshift has a relation with the dimension of the nanowires. The diameter and the length of the nanowires are both increasing as the growing stage is promoted. The quantity is obtained by the SEM observation and listed in Table 1 with the corresponding blueshift. The blueshift of PL is from 66 to 18 meV, and the nanowire diameter is from 40 to 93 nm. The blueshift of PL varies with the diameter of the nanowires. It is to prove that the blueshift of PL is directly related with the diameter of the nanowires. For comparison, the nanowires with smaller diameter and larger length are fabricated. It is realized by reducing the time for catalyst fabrication. Trace (d) is for these nanowires. Taking traces (a) and (d) for study, their diameters are close while their lengths are quite different. It can be seen that their blueshifts have a little difference. This means that the PL blueshift of the nanowires has direct relations with the diameter, rather than the length. In the nanowire growth, the diameter increases as the growing time accumulates and the PL blueshift of the nanowires decreases.

**Table 1:** PL blueshift of the nanowires with different diameters and lengths

**Nanowires for trace**	**Diameter (nm)**	**Length (nm)**	**Blueshift (meV)**
a	40	800	66
b	67	1,750	47
c	93	2,467	18
d	45	2,500	63

**Figure 5:**
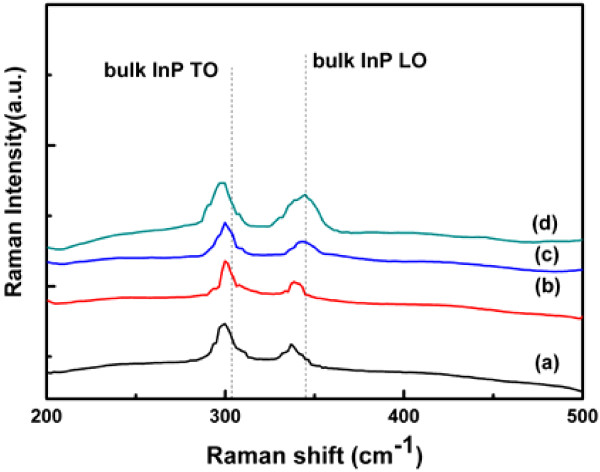
**Room-temperature Raman spectra of InP nanowires at different growing stages.** Nanowires grown for (**a**) 2, (**b**) 5, (**c**) 7, and (**d**) 15 min.

Raman spectra for nanowires at different growing stages were collected at room temperature and shown in Figure 5. Traces (a) to (d) separately correspond to the nanowires grown for 2, 5, 7, and 15 min, respectively. The peaks correspond to the transverse optical phonon (TO), and the longitudinal optical phonon (LO) is visible. As the growing time accumulates, the LO phonon gets close to the bulk InP. It means that the stress in the nanowires is being released. The full width at half maximum of the TO phonon and the LO phonon gets smaller from 2 to 5 min of growing time and gets larger after 5 min. It indicates that the quality of the crystal in InP nanowires gets better at the beginning of the nanowire growth and falls down as the growing time increases to a certain level. At the original growing stage, crystal mismatch exists between the InP nanowires and the Si substrate and the crystal quality is low. As the growing time accumulates, stress is released and the quality rises. Along with the growth, the diameter of the nanowires increases and the disfigurement on the sidewall accumulates; as a result, the crystal quality falls down.

## Conclusions

The catalyst-free MOCVD growth of the InP nanowires on Si (100) substrates is demonstrated as a dynamic process. The density of the nanowires is increasing as time accumulates. The catalyst fabricated in the nanowire growing process is caused by two reasons. One is the temperature region for indium droplet fabrication and InP nanowire growth matched together. The other is the existence of the space for accumulating TMIn and decomposed on the surface. The new nanowires fabricated in the nanowire growing process have the same crystal structure with the nanowires grown on the indium droplets. The nanowires exhibited that room-temperature photoluminescence blueshifted from the bulk zinc blende InP bandgap energy. The bandgap of the nanowires has direct relations with the diameter. In the nanowire growth, the bandgap of the nanowires can be modulated by adjusting the diameter of the nanowires. Controlling the nanowire growing time or the size of the catalyst will be possible. The crystal quality of the InP nanowires at different growing stages is a dynamic process. These results verify and supplement the research on catalyst-free InP nanowire growth.

## Abbreviations

LO, longitudinal optical phonon; MOCVD, metal organic chemical vapor deposition; PH3, phosphine; PL, photoluminescence; SEM, scanning electron microscopy; TMIn, trimethylindium; TO, transverse optical phonon.

## Competing interests

The authors declare that they have no competing interests.

## Authors’ contributions

GQM and DWZ conceived the study, participated in its design, and drafted the manuscript. Both authors carried out the experiments. GQM revised the manuscript. Both authors read and approved the final manuscript.

## Authors’ information

GQM is a professor and DWZ is an assistant professor in the State Key Laboratory of Luminescence and Applications, Changchun Institute of Optics, Fine Mechanics and Physics, Chinese Academy of Sciences, 3888 Dongnanhu Road, Changchun 130033, People’s Republic of China.
